# The emerged genotype I of Japanese encephalitis virus shows an infectivity similar to genotype III in *Culex pipiens* mosquitoes from China

**DOI:** 10.1371/journal.pntd.0007716

**Published:** 2019-09-26

**Authors:** Muddassar Hameed, Ke Liu, Muhammad Naveed Anwar, Abdul Wahaab, Anum Safdar, Di Di, Prerona Boruah, Jinpeng Xu, Xin Wang, Beibei Li, Huaimin Zhu, Mohsin Nawaz, Donghua Shao, Yafeng Qiu, Jianchao Wei, Zhiyong Ma

**Affiliations:** 1 Shanghai Veterinary Research Institute, Chinese Academy of Agricultural Science, Shanghai, PR China; 2 Department of Pathogen biology, Second Military Medical University, Shanghai, PR China; Naval Medical Research Center; Biological Defense Research Directorate, UNITED STATES

## Abstract

Japanese Encephalitis virus (JEV) is a zoonotic flavivirus that represents the most significant etiology of childhood viral neurological infections throughout the Asia. During the last 20 years, JEV genotype dominance has shifted from genotype III (GIII) to genotype I (GI). To date, the exact mechanism of this displacement is still not known. *Culex* (*Cx*.) mosquitoes are the most common species in China and play an essential role in maintaining JEV enzootic transmission cycle. In this study, we used *Cx*. *pipiens* mosquitoes from China as an *in vivo* mosquito model to explore if mosquitoes played a potential role in JEV genotype shift. We exposed female *Cx*. *pipiens* mosquitoes orally to either GI or GIII JEV strains. Midgut, whole mosquitoes, secondary organs, and salivary glands of JEV-infected mosquitoes were collected at 7 and 14 days of post infection (dpi) and subjected to measure the infection rate, replication kinetics, dissemination rate and transmission potential of the infected JEV strains in *Cx*. *pipiens* mosquitoes by 50% tissue culture infective dose assay. We found that *Cx*. *pipiens* mosquito was competent vector for both GI and GIII JEV infection, with similar infection rates and growth kinetics. After the establishment of infection, *Cx*. *pipiens* mosquitoes disseminated both JEV genotypes to secondary organs at similar rates of dissemination. A few GI-infected mosquito salivary glands (16.2%) were positive for GI virus, whereas GIII virus was undetectable in GIII-infected mosquito salivary glands at 7 dpi. However, 29.4% (5/17) and 36.3% (8/22) were positive for GI- and GIII-infected mosquito salivary glands at 14 dpi, respectively, showing an increase in JEV positive rate. No statistical difference in the transmission rate between GI- and GIII-infected mosquitoes was detected. Our experiment data demonstrated that GI and GIII viruses have similar infectivity in *Cx*. *pipiens* mosquitoes, suggesting that *Cx*. *pipiens* mosquitoes from China may not play a critical role in JEV genotype shift. Although the current data were obtained solely from *Cx*. *pipiens* mosquitoes, it is likely that the conclusion drawn could be extrapolated to the role of mosquitoes in JEV genotype shift.

## Author summary

Japanese encephalitis virus (JEV) causes encephalitis and reproductive disorder in humans and pigs respectively, causing a serious impact on public health and pig industry. In nature JEV life cycle include both vertebrates (birds and pigs) as well as invertebrates (mosquitoes). Phylogenetic studies confirmed that JEV has five geographically and epidemiologically distinct genotypes (GI-V). Genotype III (GIII) was an endemic strain in Asia, but recently genotype I (GI) has displaced GIII as the most frequently isolated virus genotype. It is unclear if mosquitoes play a role in this genotype shift or not. Both genotypes are endemic in China and primarily transmitted by different species of *Culex (Cx*.*)* mosquitoes. *Cx*. Mosquitoes are the most common species in China and their role in this genotype shift was not studied previously. In the present study, we used *Cx*. *pipiens* mosquitoes from China as an *in vivo* mosquito model and challenged with GI and GIII JEV strains to explore if mosquitoes played a potential role in JEV genotype shift. Our investigation showed that GI and GIII viruses had similar infectivity in *Cx*. *pipiens* mosquitoes which highlighted that *Cx*. *pipiens* mosquitoes from China may not play a critical role in the genotype shift. Although the current data were obtained solely from *Cx*. *pipiens* mosquitoes, it is likely that the conclusion drawn could be extrapolated to the role of mosquitoes in JEV genotype shift.

## Introduction

JEV is one of the leading encephalitis causing virus in the world [1]. According to World Health organization (WHO) more than 24 countries from South Asia and Western Pacific regions have exposed to JEV [1, 2]. JEV transmission cycle include both vertebrates (birds and pigs) as well as invertebrates (mosquitoes). Like other arboviruses, JEV is also transmitted by several *Culex* (*Cx*.), *Aedes* (*Ae*.), *Anopheles (An*.) and *Armigeres* (*Ar*.) mosquito species [3, 4]. However, *Cx*. mosquitoes have received much attention because they play a major role in transmission of JEV [4, 5]. Pigs and water birds act as an amplifying/reservoir host and later have an important role in its dispersion [6]. Usually, JEV is transmitted from infected birds/pigs to a susceptible host by mosquitoes [7]. Humans are considered dead end host of JEV infection, because humans infected with JEV seldom develop high viremia therefore, mosquitoes cannot get infection from JEV-infected persons [8].

JEV has a positive sense RNA genome belonging to *flavivirus* genus within *flaviviridae* family with three structural and seven non-structural proteins. Phylogenetic analysis indicated that it has five geographically and epidemiologically distinct genotypes (genotype I-V). Genotype III (GIII) had been the most dominant strain and source of outbreaks throughout the years. It was constantly circulating until 1990 throughout Asia, but recent studies have shown the emergence of genotype I (GI) which have displaced GIII [9–13]. According to previous data, GI diverged from Vietnam and spread towards North China, followed by Japan and Korea [14]. However, the mechanism responsible for the JEV genotype shift is unknown. Analysis of GI isolate multiplication shows that the infectivity titers after 24–48 hours post infection are significantly higher in avian and mosquito cells compared to GIII isolates. This indeed implies that high multiplicative ability of GI virus in mosquito infection may have resulted in a decreased incubation period that leads to higher GI enzootic transmission cycles and displaced GIII [15]. Another comparative study of JEV genetics reveals that this genotype shift might be due to differences in the amino acid sequences of NS5 RNA-dependent RNA polymerase between GI and GIII strains, that may help GI to achieve more efficient replication [16]. Although these previously provided data helps us to develop our understanding about JEV genetics and epidemiology, but exact mechanisms involved in GI emergence need to be elucidated.

JEV employs multiple species of mosquitoes to maintain its transmission cycle in nature [17]. In addition to the well-characterized *Cx*. *tritaeniorhynchus*, a number of other mosquito species are also competent for JEV infection. JEV has been isolated worldwidely from 17 species of *Cx*. mosquitoes, such as *Cx*. *pipiens*, *Cx*. *theileri*, *Cx*. *modestus*, *Cx*. *quinquefasciatus*, *Cx*. *fuscocephalus*, *Cx*. *annulirostris*, *Cx*. *gelidus*, *Cx*. *whitmorei*, *Cx*. *epidesmus*, *Cx*. *vishnui*, and *Cx*. *pseudovishnui*, and from 20 other mosquito species, such as *Ar*. *subalbatus*, *Ae*. *vexans*, *Ae*. *lineatopennis* and *An*. *sinensis* [18]. Several species of mosquitoes distributed in China, including *Cx*. *tritaeniorhynchus*, *Cx*. *pipiens*, *Cx*. *theileri*, *Cx*. *modestus*, *Cx*. *fuscocephalus*, *Ar*. *subalbatus*, *Ae*. *vexans* and *An*. *sinensis*, are competent vectors for JEV infection [19].

*Cx*. *pipiens* is one of the most widely distributed *Cx*. species in the world, especially in temperate regions, and lives in close contact with humans as well as animals [20]. *Cx*. *pipiens* is considered an important secondary or regional vector in certain areas such as temperate regions [18]. Both GI and GIII viruses have been isolated from *Cx*. *pipiens* [19], suggesting that *Cx*. *pipiens* is a potential vector for both genotypes transmission. Given that GI isolate shows higher infectivity than GIII isolates in mosquito cells [15], we therefore used *Cx*. *pipiens* mosquitoes from China as an *in vivo* mosquito model to examine its vector potential for GI and GIII JEV infection as well as the difference in infectivity between GI and GIII viruses to get an insight into its role in JEV genotype shift. We found that *Cx*. *pipiens* mosquitoes from China were competent vector for both GI and GIII JEV infection, with similar infection rates, growth kinetics, dissemination rates and transmission rates, showing that GI and GIII viruses have similar infectivity in *Cx*. *pipiens* mosquitoes.

## Materials and methods

### Cells and viruses

*Aedes albopictus* C6/36 cells were maintained at 28°C in SILAC^™^ RPMI 1640 (Life Technologies Ltd, Grand Island, USA) and Baby Hamster Kidney BHK-21 cells were maintained at 37°C in Dulbecco Modified Eagle media DMEM (Life Technologies) supplemented with 10% heat-inactivated fetal bovine serum (FBS), 100 IU of penicillin and streptomycin per ml for JEV production [5, 21–23]. JEV GI SH7 strain (GeneBank accession no MH753129) was isolated from *Cx*. *tritaeniorhynchus* in 2016 and GIII SH15 strain (GeneBank accession no. MH753130) was isolated from *An*. *sinensis* in 2016. Both strains were passaged fewer than seven times in cultured cells, including three passages for plaque purification and one passage on C6/36 cells for mosquito infection. The 50% tissue culture infective dose (TCID_50_) were determined on BHK-21 cells [23]. Fresh virus suspensions were used in whole experiment.

### Mosquitoes rearing and infections

*Cx*. *pipiens* mosquitoes used for this experiment were provided by Dr. Zhu Huiman from Second Military Medical University Shanghai, PR China. Mosquitoes were maintained on 10% sucrose ad libitum solution and kept at 28°C with 70% to 80% relative humidity and 12-h-light–12-h-dark photoperiod in cages according to standard conditions [24, 25]. For per os infection, 5–7 days-old female mosquitoes were deprived of sugar and water for 48 and 24 hours, respectively. Viremic blood meals were prepared by mixing virus stocks with defebrinated mice blood and delivered through Hemotek membrane feeding apparatus (Discovery Workshop) and cotton pledget for one hour. Mosquitoes were cold anesthetized on ice prior to sorting fully engorged mosquitoes. 5–6 engorged mosquitoes were immediately collected to determine the quantities of viruses ingested through the blood meals. Titers of viremic blood meals and engorged mosquitoes are summarized in Table 1.

**Table 1:** JEV titers of viremic blood meals and engorged mosquitoes.

JEV strains	GI (SH7)	GIII (SH15)	*p* value*
Viremic blood meals (log TCID_50_/ml)	8.30±0.30	8.65±0.15	0.4063
Engorged mosquitoes (log TCID_50_/ml)	4.90±0.80	4.78±0.46	0.6255

*, tested by Student’s *t*-test.

### Sample collection and JEV titration

After oral feeding, engorged mosquitoes were randomly divided into different groups (*n*>13) and held for extrinsic incubation period. At 7 and 14 dpi samples were collected in DMEM and JEV titration was performed by TCID_50_ assay [4, 26, 27] to determine the infection rate, growth kinetics, dissemination rate, and transmission rate [28, 29]. Infection rate was defined as the number of mosquitoes with infectious JEV in the midgut divided by the total number of engorged mosquitoes tested. Dissemination rate was defined by the detection of infectious JEV from homogenized secondary organs (legs, wings and heads) among mosquitoes with positive midguts. Transmission rate was defined as the number of mosquitoes with infectious JEV in the salivary glands divided by the total number of mosquitoes with positive midguts. Mosquitoes were caught by mechanical aspiration from cages and anesthetized using ice. Dissection of individual mosquito was conducted under stereomicroscope using dissecting needles to collect midgut for infection rate [30], secondary organs for dissemination rate, and salivary glands for transmission rates, as described previously by Coleman et al [30]. Whole mosquitoes were used to assess JEV growth kinetics. To avoid cross-contamination of virus across the midgut, secondary organs and salivary glands, these organs were dissected carefully using different dissecting needles and dipped in 75% ethanol followed by distal water [27]. Quantification of each sample were performed by TCID_50_ assay on BHK-21 cells according to previously described method [4, 23].

### Statistical analysis

Student *t*-test or Fisher’s Exact test was performed for statistical analysis. A *p* value of <0.05 was considered significant. Graph Pad Prism software (version 7) was used for all statistical analysis.

## Results

### Efficiency in the establishment of JEV infection in midgut of *Cx*. *pipiens* mosquitoes from China

**Fig 1:**
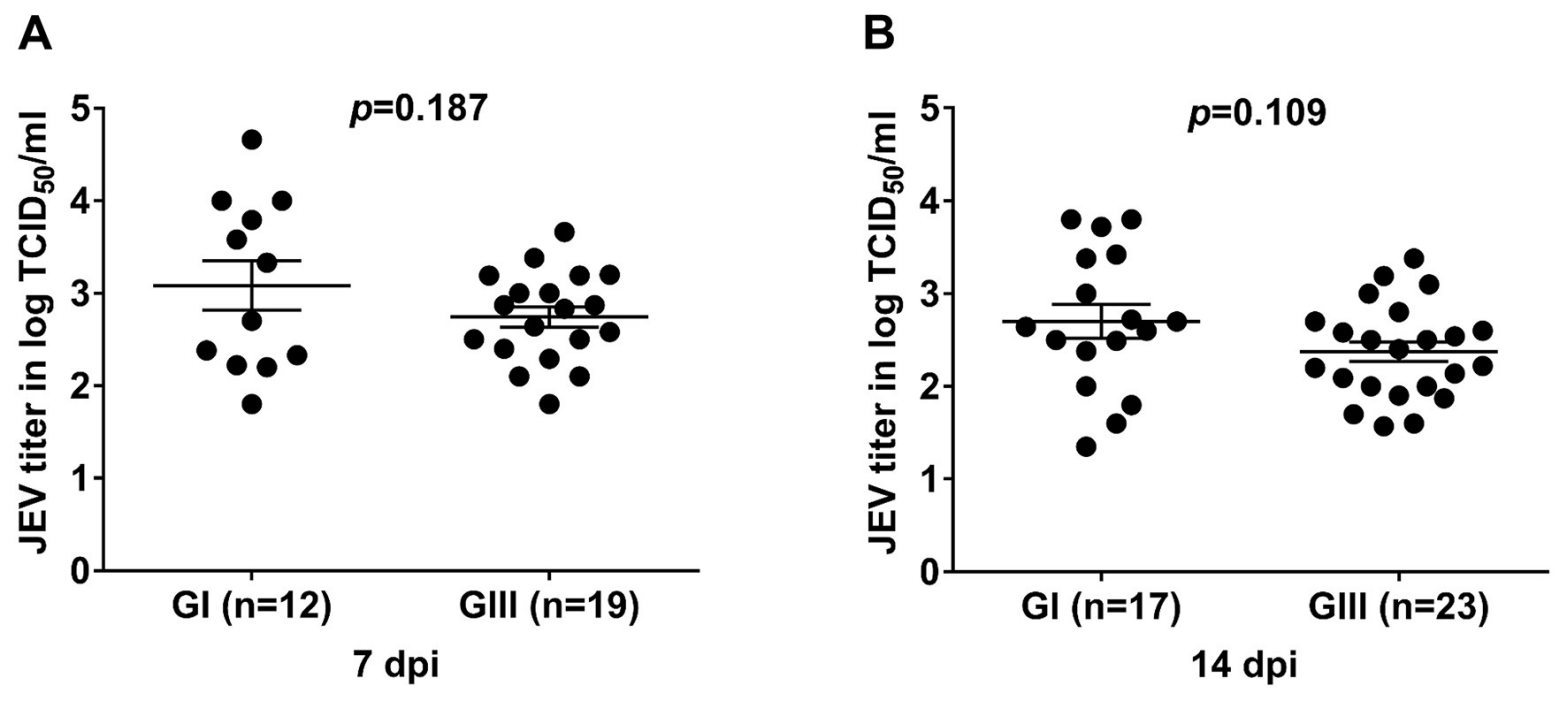
Viral titers in midgut of JEV-infected mosquitoes. *Cx*. *pipiens* mosquitoes were orally infected with GI or GIII viruses and midguts of the infected mosquitoes were collected at 7 dpi (A) and 14 dpi (B) for measurement of JEV titers by TCID_50_ assay. *n*, the numbers of mosquitoes tested positive for JEV. The *p* values were generated by Student *t*-test between GI- and GIII-infected groups. A *p* value of <0.05 was considered significant.

*Cx*. *pipiens* mosquitoes from China were used as a *in vivo* model to examine its vector potential for GI and GIII JEV infection as well as the difference in infectivity between GI and GIII viruses. Midgut is one of mosquito tissues used for analysis of the vector permissive to flavivirus infection [31–34]. Therefore, we determined the replication titers in midguts of *Cx*. *pipiens* to explore its vector competence for different JEV genotype infection. The mosquitoes were orally infected with GI and GIII viruses and six of mosquitoes for each group were randomly collected from the engorged mosquitoes immediately after blood feeding for detection of JEV titers. No significant difference in JEV titers was observed between GI- and GIII-infected groups (Table 1). JEV replication in the midgut of JEV-infected mosquitoes was determined by TCID_50_ assay at 7 and 14 dpi. Infection rates in GI-infected group were 42.8% (12/28) at 7 dpi and 37.7% (17/45) at 14 dpi, which were similar to those 57.5% (19/33) at 7 dpi and 44.2% (23/52) at 14 dpi in GIII-infected group (Table 2). At 7 dpi, similar replication titers (*p* = 0.187) between GI-infected group (3.083logTCID_50_ /ml) and GIII-infected group (2.748logTCID_50_ /ml) were observed (Fig 1A). However, the replication titers were slightly declined at 14 dpi in both JEV-infected groups, but no significant difference (*p* = 0.109) in replication titers were detected between GI-infected group (2.70logTCID_50_ /ml) and GIII-infected group (2.37logTCID_50_ /ml) (Fig 1B). Cumulatively, these data indicated that *Cx*. *pipiens* mosquito was competent vector for both GI and GIII JEV infection, with similar infection rate and replication titers.

**Table 2:** Summary of the infection, dissemination and transmission rates of JEV infected mosquitoes.

JEV strains		GI (SH7)	GIII (SH15)	*p* value*
Infection rate	7 dpi	42.8% (12/28)	57.5% (19/33)	0.3087
	14 dpi	37.7% (17/45)	44.2% (23/52)	0.5422
Dissemination rate	7 dpi	27.2% (3/11)	31.5% (6/19)	>0.9999
	14 dpi	23.5% (4/17)	34.7% (8/23)	0.6989
Transmission rate	7 dpi	16.6% (2/12)	0% (0/13)	0.2200
	14 dpi	29.4% (5/17)	36.3% (8/22)	0.7401

*, tested by Fisher’s Exact test.

### Replication kinetics of JEV in *Cx*. *pipiens* mosquitoes

**Fig 2:**
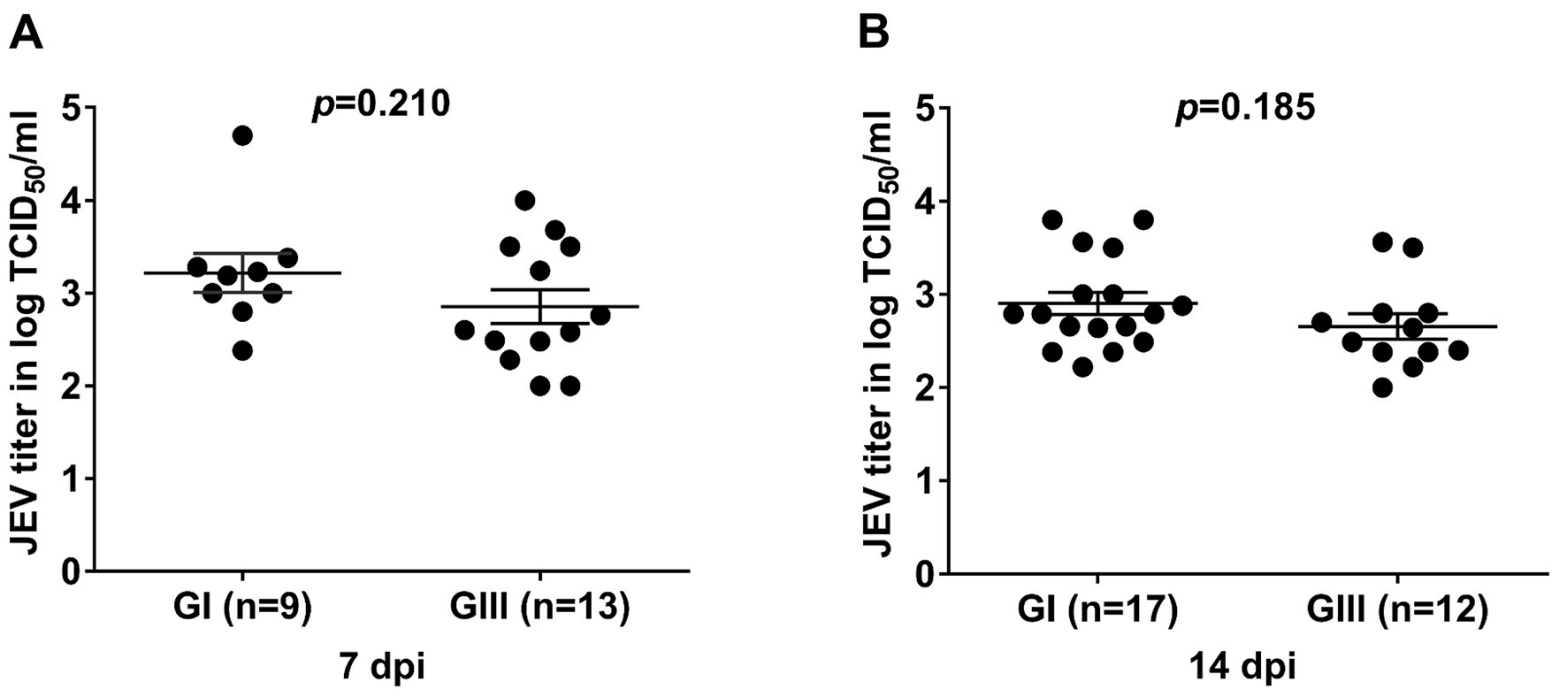
Viral titers in whole mosquitoes of JEV-infected mosquitoes. *Cx*. *pipiens* mosquitoes were orally infected with GI or GIII viruses and whole mosquitoes were collected at 7 dpi (A) and 14 dpi (B) for measurement of JEV titers by TCID_50_ assay on BHK-21 cells. *n*, the numbers of mosquitoes tested positive for JEV. The *p* values were generated by Student *t*-test between GI- and GIII-infected groups. A *p* value of <0.05 was considered significant.

JEV replicates in various tissues of mosquitoes [33, 35, 36], we therefore determined replication kinetics in whole mosquitoes infected with GI or GIII JEV. Following oral infection, the *Cx*. *pipiens* mosquitoes were collected at 7 and 14 dpi and JEV titers were measured by TCID_50_ assay. Among 16 and 27 GI-infected mosquitoes collected at 7 and 14 dpi, 9 and 17 were tested positive for JEV, respectively. While out of 20 and 25 GIII-infected mosquitoes collected at 7 and 14 dpi, 13 and 12 were tested positive for JEV, respectively. The replication titers in the JEV-positive mosquitoes were further compared between GI- and GIII-infected groups. No significant difference in replication titers between GI- and GIII-infected mosquitoes were detected at both 7 and 14 dpi. As shown in Fig 2, the replication titers in GI-infected group were 3.21logTCID_50_ /ml at 7 dpi (*n* = 9), which was statistically similar (*p* = 0.2107) with that (2.85logTCID_50_ /ml) in GIII-infected group (*n* = 13) (Fig 2A). At 14 dpi, the replication titer (2.90logTCID_50_ /ml) in GI-infected group (*n* = 17) was also relatively higher than that (2.65logTCID_50_ /ml) in GIII-infected group (*n* = 12), but no significant difference was detected (*p* = 0.1859) (Fig 2B). Overall, these results suggested that the GI and GIII JEV replicated with similar kinetics in *Cx*. *pipiens* mosquitoes.

### Dissemination of JEV in *Cx*. *pipiens* mosquitoes

To determine whether JEV-infected mosquitoes could disseminate virus to secondary organs, *Cx*. *pipiens* mosquitoes were infected orally with GI or GIII JEV and the secondary organs including legs, wings and heads from JEV-infected mosquitoes that were tested positive for JEV in midguts were collected at 7 and 14 dpi for detection of JEV presence. No statistical difference in the dissemination rates between GI- and GIII-infected mosquitoes was tested at both 7 dpi (Fisher’s Exact test, *p*>0.9999) and 14 dpi (Fisher’s Exact test, *p* = 0.6989). As shown in Table 2, the dissemination rate of GI-infected mosquitoes was 27.2%, which was not significantly different to that (31.5%) of GIII-infected mosquitoes at 7 dpi. Similar results were observed at 14 dpi between GI- (23.5%) and GIII- (34.7%) infected mosquitoes. These results suggested that *Cx*. *pipiens* mosquitoes disseminated JEV to secondary organs after the establishment of infection, with similar dissemination rate between GI and GIII viruses.

### Viral load in salivary glands of JEV infected *Cx*. *pipiens* mosquitoes

**Fig 3:**
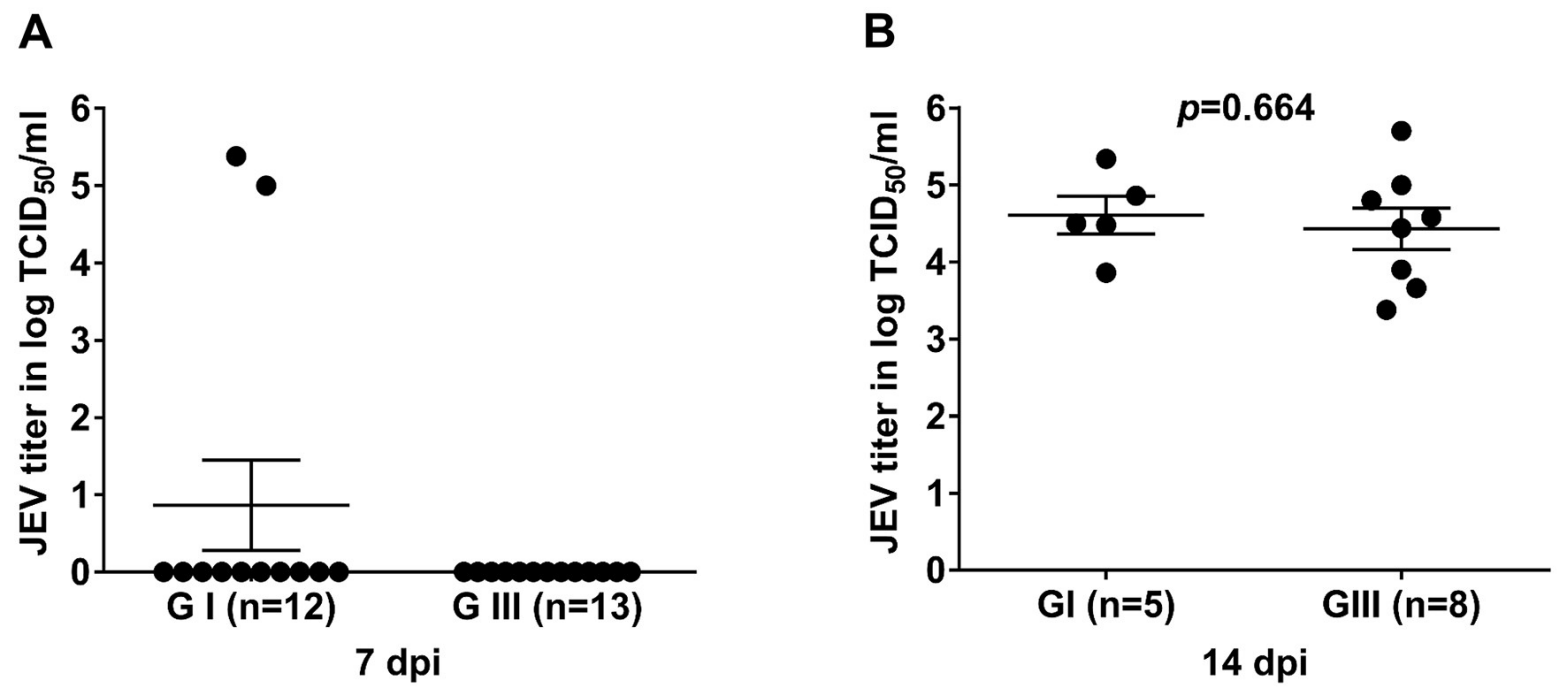
Viral titers in salivary glands of JEV-infected mosquitoes. *Cx*. *pipiens* mosquitoes were orally infected with GI or GIII viruses and salivary glands of the infected mosquitoes were collected at 7 dpi (A) and 14 dpi (B) for measurement of JEV titers by TCID_50_ assay on BHK-21 cells. *n*, the numbers of mosquitoes tested positive for JEV in midguts. The *p* values were generated by Student *t*-test between GI- and GIII-infected groups. A *p* value of <0.05 was considered significant.

Saliva plays crucial role in transmission of flaviviruses [27, 37, 38], we therefore determined the viral load in salivary glands of JEV-infected mosquitoes to compare the potentials of *Cx*. *pipiens* mosquitoes in JEV transmission. The salivary glands of JEV-infected mosquitoes that were tested positive for JEV in midguts were subjected to analysis of viral load by TCID_50_ assay. Out of 12 GI-infected mosquitoes tested, two showed positive for JEV in the salivary glands at 7 dpi, whereas no mosquito was detected positive in the salivary gland among 13 GIII-infected mosquitoes tested (Fig 3A, Table 2). However, 29.4% (5/17) and 36.3% (8/22) were positive for GI and GIII viruses in the salivary glands at 14 dpi, respectively; showing an increase in JEV positive rate, but no significant difference was detected between GI- and GIII-infected groups (Table 2). At 14 dpi, JEV titer in the salivary glands of GI-infected mosquitoes was 4.60logTCID_50_/ml that was not significantly different (*p* = 0.6648) from that (4.33logTCID_50_/ml) in the salivary glands of GIII-infected mosquitoes (Fig 3B). It has previously been shown that virus must go through the midgut barrier and then spread to salivary glands. Therefore, the time of virus detected in salivary glands was later than the time of detection in midgut [39, 40]. Altogether these results indicated that the *Cx*. *pipiens* mosquitoes had potential for transmission of both genotype viruses, with similar transmission rates.

## Discussion

In recent years, multiple reports indicated that GI JEV has taken over GIII, as the most frequently isolated strain in a number of Asian countries [41–43]. The mechanism of this shifting is still unclear. A previous observation describes that GI strain has superior multiplication kinetics in C6/36 cells as compared to GIII strain [15], implying that mosquitoes may play a potential role in JEV genotype shift. *Cx*. *pipiens* mosquito is one of the most common and high density *Cx*. species [44–46] and lives in close contact with humans as well as animals with blood-feeding behavior [20, 47]. In addition, *Cx*. *pipiens* mosquito is easy to be reared and experimentally infected with flavivirus in laboratorial condition [48–50]. In response to JEV infection, *Cx*. *pipiens* shows susceptibility similar to *Cx*. *tritaeniorhyncus* and has been considered as an effective laboratory vector for JEV infection [51]. Therefore, we used *Cx*. *pipiens* mosquito as an *in vivo* mosquito model to explore whether mosquito could play a role in JEV genotype shift. Although *Cx*. *tritaeniorhyncus* is the primary vector for JEV transmission, other species of *Cx*. mosquitoes, such as *Cx*. *pipiens*, are considered important secondary or regional vectors in certain areas such as temperate regions [18]. It has been reported that the increasing JE cases are observed in the area with very less density of *Cx*. *tritaeniorhyncus* (1%) and high population of *Cx*. *pipiens* (>60%) [45], suggesting that *Cx*. *pipiens* other than *Cx*. *tritaeniorhyncus* may play a major role in JEV transmission in certain areas. In addition, *Cx*. *pipiens* feeds mostly on birds (83%) [49, 52] that act as the reservoir and amplifying hosts for maintaining JEV transmission cycle and have been speculated to be involved in the increased JE cases and genotype shift [45, 53].

We have previously compared the replication efficiency of 3 GI isolates with 4 GIII isolates in mosquito cells and found no significant difference in replication efficiency between GI and GIII isolates tested, suggesting similar replication efficiency between GI and GIII isolates in mosquito cells [53]. Based on these findings, we selected one GI isolate (SH7 strain) and one GIII isolate (SH15 strain) to compare the replication efficiency in *Cx*. *pipiens* mosquitoes. Both strains were isolated from mosquitoes in 2016 and have replication kinetics similar to the average replication kinetics of their respective genotype isolates in mosquito, swine and avian cells [53]. Therefore, we considered that these two strains could be representative of their respective genotypes.

We demonstrated experimentally that *Cx*. *pipiens* mosquito was competent vector for both GI and GIII infection. The infection rates and growth kinetics in the orally infected *Cx*. *pipiens* mosquitoes showed no significant difference between GI- and GIII-infected groups. After development of infection, *Cx*. *pipiens* mosquitoes disseminated both JEV genotypes to secondary organs at similar dissemination rate. GI and GIII were detectable in the salivary glands with 29.4% to 36.3% positive rate at 14 dpi, suggesting a potential of *Cx*. *pipiens* mosquitoes for transmission of both genotype viruses. However, no significant difference in the transmission rates between GI- and GIII-infected mosquitoes was detected. As whole, our experiment data demonstrated that GI and GIII viruses have similar infectivity in *Cx*. *pipiens* mosquitoes, suggesting that *Cx*. *pipiens* mosquitoes from China may not play a critical role in JEV genotype shift. However, this conclusion was generated by the use of a single representative JEV strain from each genotype, further studies with more different GI and GIII JEV strains should be conducted to confirm this conclusion.

A previous *in vitro* observation indicates that the infectivity titers of GI isolate after 24–48 hours post infection are significantly higher in mosquito cells compared to GIII isolates [15]. This observation is partially in contrast with our findings that no significant difference in infectivity titers between GI and GIII strains was observed in *Cx*. *pipiens* mosquitoes. However, our findings are consistent with a previous *in vivo* observation [5], in which similar infection rate, dissemination rate and transmission rate are observed between GI- and GIII-infected North American *Cx*. *quinquefasciatus* mosquitoes. Our data together with the previous *in vivo* observations, suggested that *Cx*. species are competent vectors for both GI and GIII JEV infection with similar infectivity and that *Cx*. *pipiens* mosquitoes may not play a critical role in JEV genotype shift. This conclusion is further supported by Wispelaere et al’s observation [4], in which they analyze the vector competence of European *Cx*. *pipiens* mosquitoes for GIII and genotype V (GV) JEV infection and noted similar infectivity between GIII- and GV- infected *Cx*. *pipiens* mosquitoes.

An interesting observation from our experiments was the earlier viral load detected in salivary glands of GI-infected *Cx*. *pipiens* mosquitoes. Out of 12 GI-infected mosquitoes tested, two showed positive for JEV in the salivary glands at 7 dpi, whereas no mosquito was detected positive in the salivary gland among 13 GIII-infected mosquitoes tested. Saliva plays crucial role in JEV transmission. The earlier viral load in salivary glands of GI-infected mosquitoes could be taken into account as a potential factor when dissecting the mechanisms responsible for JEV genotype shift.

Genetic drift during systemic arbovirus infection of mosquitoes randomly generates tissue and saliva specific progeny arbovirus [40, 54–56]. For example, mutant progeny of West Nile virus (WNV) is transiently detected in the saliva of infected individual mosquito between feeding episodes. The mutant WNV has advantage in competitive fitness relative to the reference WNV in *Cx*. *quinquefasciatus* mosquitoes, but becomes extinct in some individual mosquito during competitive fitness assays [57]. We did not know whether the earlier viral load detected in the salivary glands of GI-infected mosquitoes was the progeny virus specific to salivary glands generated by genetic drift or attributable to the enhanced vector fitness generated by convergent evolution. Future studies with virus isolation and genome sequencing under different time intervals should be conducted to determine the reasons responsible for the earlier viral load detected in the salivary glands of GI-infected *Cx*. *pipiens* mosquitoes, which could be useful for elucidating the mechanisms of JEV genotype shift.

JEV enzootic transmission cycle is maintained by both vertebrates (wild birds and pigs) and invertebrates (mosquitoes) [3, 4, 17, 58]. In addition to mosquitoes, pig serves as a major amplifying host of JEV in Asia, especially in China pork industry has grown exponentially (87%) within the last twenty years [47] and also close proximity of pig breeding farms to suburban areas increases the risk of JE cases [59]. Birds serve as an amplifying/reservoir host of JEV. Previous studies reported that avian species can develop viremia either with natural exposure or by challenging in laboratory [17, 60–62]. In 2009, Saito et al. suggested that wild ducks can play a role in JEV reservoir in Hakkaido, Japan [63]. These findings further supported by Yang et al., where he had reported 84%-88.5% sero-prevalence of JEV in different wild birds including ducks [64]. A most recent study in Korea demonstrated that distribution of wading birds and the incidence of JE cases are correlated [45]. Recently, our lab also reported that GI replicates more efficiently than GIII in avian and porcine cells, particularly in avian cells with titers reaching 22.9−225.3 fold higher than GIII. In addition, GI-inoculated ducklings developed higher viremia titers and showed a relatively longer viremic duration than GIII-inoculated ducklings [53]. These reports suggest that pigs/birds may have some important role in JEV genotype shift that need to be explored in future.

In addition to JEV hosts, phylogenetic studies suggest that the mechanism of JEV genotype shift might be due to amino acid variations between GI and GIII viral proteins, especially the variation in JEV envelope protein that plays major roles in mediating virus entry and pathogenicity [9, 15, 65], and the variation in JEV nonstructural protein 5 that plays essential roles in methylation of the 5’ RNA cap structure, viral replication and antagonization of the interferon response [66]. These amino acid variations alone or in combination with variations in other proteins or genomes may lead to GI viruses with increased host fitness and enhanced multiplicative ability in hosts such as birds and pigs and eventually displacement of GIII as a dominant genotype. This hypothesis is currently under investigation in our laboratory.

In conclusion, we compared the infectivity of GI and GIII viruses in *Cx*. *pipiens* mosquitoes and found that *Cx*. *pipiens* mosquito is competent vector for both GI and GIII JEV infection, with similar infection rate and growth kinetics. *Cx*. *pipiens* mosquitoes were able to disseminate both JEV genotype to secondary organs at similar level of dissemination. Both JEV genotypes were detectable in the salivary glands of infected mosquitoes at similar transmission rate, suggesting the potential of *Cx*. *pipiens* mosquitoes for transmission of both genotype viruses. Our experiment data demonstrated that GI and GIII viruses had similar infectivity in *Cx*. *pipiens* mosquitoes, suggesting that *Cx*. *pipiens* mosquitoes from China may not play a critical role in JEV genotype shift. However, this conclusion was generated by the use of a single representative JEV strain from each genotype, further studies with more different GI and GIII JEV strains should be conducted to confirm this conclusion. Although the current data were obtained solely from *Cx*. *pipiens* mosquitoes, it is likely that the conclusion drawn could be extrapolated to the role of mosquitoes in JEV genotype shift.
